# In This Issue: Volume 117, Issue 11, November 2026

**DOI:** 10.1111/cas.70545

**Published:** 2026-11-02

## Nicotinic Acid Restriction Enhances the Therapeutic Benefit of NAMPT Inhibition in Small‐Cell Lung Cancer Models

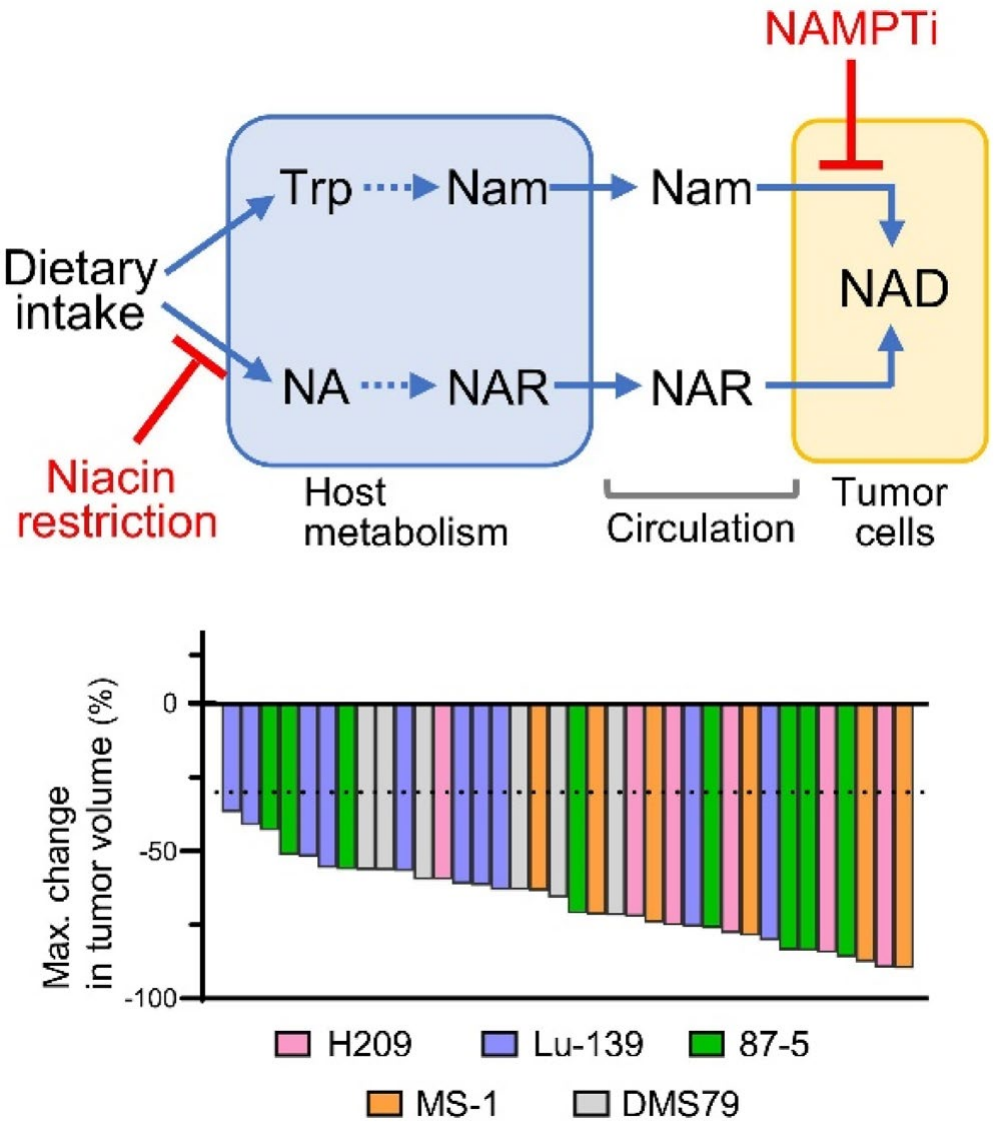

Small‐cell lung cancer (SCLC) is a fast‐growing type of lung cancer that can spread quickly. Although chemotherapy can be effective at first, the cancer often develops resistance, leaving few treatment options. This highlights the need for development of new therapeutic strategies.

Cancer cells exhibit varied characteristics and can become highly dependent on specific molecules for their growth. One such molecule is nicotinamide adenine dinucleotide (NAD), which cells need to produce energy and carry out many important functions. One of the main ways they make NAD involves a protein called nicotinamide phosphoribosyltransferase (NAMPT). Drugs that block NAMPT have therefore been explored as potential cancer treatments. However, blocking this pathway alone does not completely stop SCLC cells from making NAD. The cells can use another route that is supported by nicotinic acid, a water‐soluble vitamin that can be obtained from dietary sources.

In this context, researchers tested whether blocking both routes at the same time would make it difficult for SCLC cells to survive. For this, they combined a NAMPT‐blocking drug with a diet lacking nicotinic acid and tested the treatment in several mouse models of SCLC. They also used models derived from chemotherapy‐resistant tumors.

Notably, the combination treatment was much more effective than the NAMPT‐blocking drug alone. It caused tumors to shrink in all patient‐derived models tested, including those from chemotherapy‐resistant cancers. When compared with the commonly used chemotherapy drugs cisplatin and etoposide, the combination produced greater tumor reduction in most of the models tested. The treatment also appeared to be better tolerated than chemotherapy. Mice receiving the combination had less weight loss and did not show the decrease in platelets observed with chemotherapy. However, the treatment temporarily reduced white blood cells, which recovered after treatment.

Overall, the study suggests that targeting both sources of NAD may be a promising approach for treating SCLC, particularly in cancers that no longer respond well to chemotherapy.

https://onlinelibrary.wiley.com/doi/full/10.1111/cas.70486

## Evidence That the Protein Phosphatase Activity of PTEN Contributes to Embryonic Development and Tumor Suppression

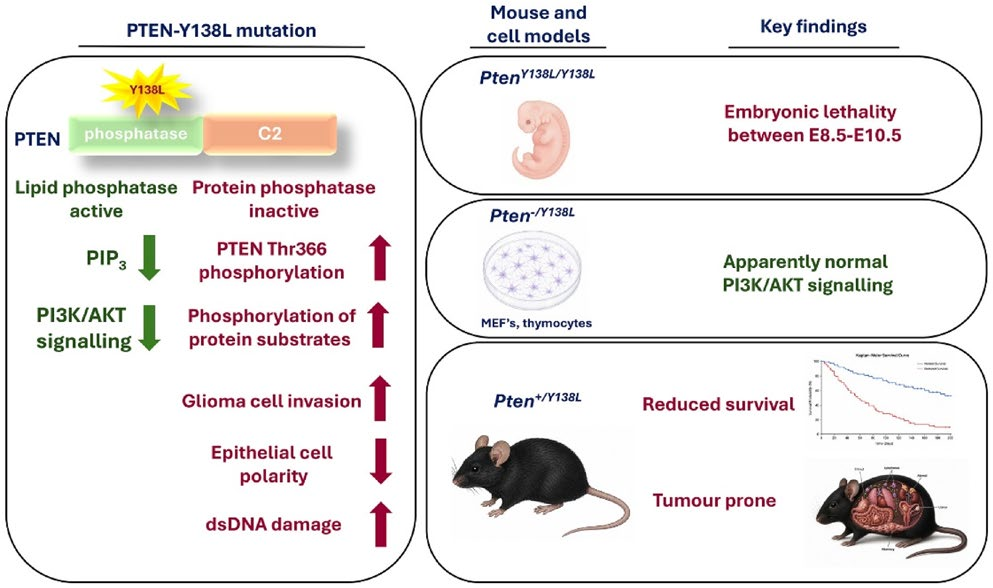

Phosphatase and tensin homolog deleted on chromosome 10 (PTEN) is a crucial tumor suppressor that keeps cells from growing out of control. It mainly acts on lipid molecules, removing phosphates from them, which is its primary tumor‐suppressive mechanism. PTEN has also been shown to dephosphorylate protein substrates including auto‐dephosphorylating itself. However, identifying the physiological significance of PTEN's protein phosphatase activity remains a challenge.

To investigate this, researchers created mice with a specific mutation in PTEN called Y138L. This change removes PTEN's ability to act on proteins but leaves its lipid phosphatase activity intact. This allowed the researchers to study what happens when PTEN loses only its protein‐related function. The results were surprising. Mice in which both PTEN genes carried this change could not develop normally and died before birth. Mice in which only one PTEN gene carried the change survived but had shorter lifespans and were more likely to develop tumors and other abnormal tissue growths in several organs, including the lymph nodes, digestive system, breast tissue, prostate, and uterus. Importantly, PTEN's lipid phosphatase activity was still able to regulate the PI3K–AKT pathway in these mice, showing that reducing AKT activity alone was not enough to provide all of PTEN's tumor suppressor effects. However, the altered PTEN was still able to prevent T‐cell lymphoma, suggesting that its lipid‐related function can be sufficient to protect against some cancers.

Overall, the study indicates that PTEN protects the body in two ways, by controlling lipid‐based growth signals and by acting on proteins, which may include itself, inside cells. Both functions appear to be important for PTEN's full role in normal development and cancer prevention. These findings could eventually help researchers develop more precise treatments for cancers in which PTEN is defective.

https://onlinelibrary.wiley.com/doi/full/10.1111/cas.70476

## Real‐World Safety and Efficacy of Pola‐R‐CHP in Previously Untreated DLBCL: POLASTAR Interim Analysis of 500 Patients

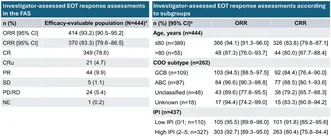

Diffuse large B‐cell lymphoma (DLBCL) is a fast‐growing blood cancer that can often be treated successfully, but some people do not respond to treatment or their cancer may return. Polatuzumab vedotin plus rituximab, cyclophosphamide, doxorubicin, and prednisolone (Pola‐R‐CHP) is a newer treatment option that has shown benefits in clinical trials. However, clinical trials do not always reflect everyday medical care, where patients may be older, frailer, or have different health conditions. Because there is limited information on how Pola‐R‐CHP performs in routine care, the POLASTAR study was designed to evaluate its effectiveness and safety in Japanese patients with DLBCL. This multicenter, prospective observational study followed patients receiving Pola‐R‐CHP in routine medical practice.

The study followed 485 people in Japan who received Pola‐R‐CHP as their first treatment for DLBCL across 71 medical centers. The study included 60 people over 80 years old, providing useful information about treatment in older adults. Among evaluable patients, 93% of patients responded to treatment, and 83% had no detectable signs of cancer at the end of treatment. Responses remained high in people over 80, even though many received lower doses of some chemotherapy medicines. Severe side effects occurred in 47% of patients overall and 57% of those over 80. The most common problems included low blood cell counts and lung infections. Five patients died following adverse events, and about 7% stopped treatment with polatuzumab because of side effects.

Together, these findings suggest that Pola‐R‐CHP can provide strong treatment responses while remaining manageable for a broad range of people with newly diagnosed DLBCL, including older adults who may not have been represented in earlier clinical trials. The results also highlight the importance of close monitoring and appropriate dose adjustments for very old or frail patients. Longer follow‐up will show how long the treatment benefits last and whether lower chemotherapy doses affect long‐term outcomes. Overall, POLASTAR adds valuable real‐world evidence on the use of Pola‐R‐CHP in routine care in Japan.

https://onlinelibrary.wiley.com/doi/full/10.1111/cas.70484

